# Long non-coding RNA LNC_000641 regulates pseudorabies virus replication

**DOI:** 10.1186/s13567-021-00922-0

**Published:** 2021-03-25

**Authors:** Linlin Fang, Yanni Gao, Xing Liu, Juan Bai, Ping Jiang, XianWei Wang

**Affiliations:** 1 Key Laboratory of Animal Diseases Diagnostic and Immunology, Ministry of Agriculture, MOE Joint International Research Laboratory of Animal Health and Food Safety, College of Veterinary Medicine, Nanjing Agricultural University, Nanjing, 210095 China; 2 Jiangsu Co-Innovation Center for the Prevention and Control of Important Animal Infectious Diseases and Zoonoses, Yangzhou University, Yangzhou, 225009 China

**Keywords:** Pseudorabies virus, Long non-coding RNAs (lncRNAs), Anti-viral activity, IFN-alpha, JAK/STAT1

## Abstract

Long non-coding RNAs (lncRNAs) are a new arm of gene regulatory mechanism as discovered by sequencing techniques and follow-up functional studies. The lncRNAs regulation of pseudorabies virus (PRV) infection has rarely been reported so far. Using RNA sequencing analysis, 225 lncRNAs with significant altered expressions in 3D4/21 cells infected with PRV (ZJ01) were identified. Five lncRNAs upregulated in PRV-infected cells were verified in cells infected with different PRV strains by qRT-PCR. By down- and up-regulation of lnc641, the accelerating effect of lnc641 on PRV replication was confirmed. Furthermore, we found that lnc641 regulated PRV replication by inhibiting the JAK-STAT1 pathway. This study suggests that lnc641 could be a new host factor target for developing antiviral therapies against PRV infection.

## Introduction

Pseudorabies virus (PRV), known as Aujeszky’s disease virus or suid herpesvirus 1, is a member of the *alphaherpesvirinae* subfamily and threatens pig production [1]. PRV is a highly infectious and lethal pathogen in pigs responsible for Aujeszky’s disease, which causes abortions and stillbirths in sows, central nervous system disorders in young piglets, and respiratory disease in older pigs. PRV genome is a double-stranded DNA with a length of 142 334 bp [2, 3]. The mature virion, or infectious viral particle, consists of four morphologically distinct structural components: the central core contains the linear double-stranded DNA genome of the virus; the DNA is enclosed within a protective icosahedral capsid to form a nucleocapsid; the capsid is embedded in a protein matrix known as the tegument; finally, the tegument is surrounded by the envelope, a lipid membrane containing several viral glycoproteins [4, 5]. PRV infection impairs interferon (IFN) signaling to establish persistent infection in host cells, by suppressing IFN-induced upregulation of STAT1 phosphorylation and various interferon-stimulated genes (ISGs) [6].

Host genome DNA sequencing is of importance for understanding host evolution, disease origin, and the interplay between environment and heredity. The emergence of high-throughput sequencing technologies has had the greatest impact on the expanding world of non-coding RNAs. The first transcriptome analyses led to the unexpected discovery that while most of the genome is transcribed, only 2% of the genome is transcribed into mRNAs encoding proteins. It was apparent that the majority of the genome was transcribed into noncoding RNAs [7]. Among the noncoding genome, long noncoding RNAs (lncRNAs) constitutes a particularly rich category. lncRNA is a class of transcripts with more than 200 bp in length without encoding possibility in eukaryotes [8, 9]. Some estimates suggest that the human genome contains more than 90 000 genes and approximately 60 000 of them are lncRNAs, while other estimates suggest that the number of lncRNA genes could reach closer to 200 000 [9].

LncRNAs play important roles in many biological processes. The roles of lncRNAs in viral infections have been documented [10, 11]. Some differentially expressed lncRNAs regulate inflammatory innate responses and pathogen evasion or survival during host–pathogen interactions [12–14]. Negative regulator of antiviral response (NRAV) is a lncRNA that is downregulated by various viruses including influenza virus, sendai virus, muscovy duck reovirus, and herpes simplex virus [15]. Overexpression of NRAV increases virus replication whereas knockdown of NRAV has an opposite effect.

The innate immune response is a host first line of defense against invading viruses [16]. Type I interferons (IFNs), primarily IFN-α/β, are produced by host cells as “early” antiviral agents [17, 18] and are recognized as a critical part of the host innate immune response to virus infection. Type I IFNs bind to their receptors to activate molecules downstream Janus kinase-signal transducer and activator of transcription (JAK–STAT) signaling that consequently initiates the transcription of ISGs, including noncoding transcripts, which exert a broad spectrum of antiviral effects [6]. In general, the binding of IFN-α/β to their receptors results in the cross-phosphorylation of Janus kinases (Jaks) at tyrosines, which provides docking sites for signal transducers and activators of transcription (Stats) leading to stat phosphorylation. The phosphorylated stats (pStats) then dissociate from the receptor, dimerize and translocate into the nucleus to regulate downstream gene expression [19]. LncRNAs such as THRIL [12] lincRNA-Cox2 and Lethe [20] have been shown to regulate gene expression in innate immune cells. LncRNAs are emerging as critical regulators of both innate and adaptive immunity [21]. These studies suggest that lncRNAs play a crucial role in virus pathogenesis. However, the regulation by lncRNAs of PRV infection is still not well known.

In the present study, using RNA sequencing analysis, we showed that the expression of 225 lncRNAs was significantly altered in 3D4/21cells infected with PRV. According to an analysis of differential expression between mock-infected and PRV-infected cells, 126 host lncRNAs were significantly upregulated and 99 host lncRNAs were significantly downregulated in the latter group.

## Materials and methods

### Cells, viruses and reagents

Porcine alveolar macrophages cells (3D4/21, ATCC® CRL-2843™), porcine kidney cells (PK-15, ATCC® CCL-33™), porcine testis cells (ST, ATCC® CRL-1746™) were stored in the laboratory. 3D4/21 cells were cultured in RPMI-1640 medium (Gibco, USA) supplemented with 10% fetal bovine serum (FBS; LONSERA) and 0.1 mM non-essential amino acid (NAA), PK-15 cells and ST cells were cultured in Dulbecco’s modified Eagle’s medium (Corning, USA) supplemented with 10% fetal bovine serum (FBS; LONSERA) at 37 °C in a humidified atmosphere containing 5% CO_2_. Two PRV strains (ZJ01 and LA) were used. PRV was proliferated in PK-15 cells and was stored at − 80 °C. The highly pathogenic PRV strain ZJ01, which is maintained in our laboratory, was used for all experiments. The PRV strain LA was also used, as specifically mentioned by name (LA, a classical strain).

Anti-PRV gB-protein monoclonal antibody (1B1, prepared and stored in our laboratory), anti-GAPDH antibody (Proteintech, USA), anti-P-STAT1 antibody (Cell Signal Technology, USA), anti-STAT1 antibody (Cell Signal Technology, USA), anti-p-JAK1 antibody (Affinity, USA) and anti-JAK1 antibody (Affinity, USA) were used in the study.

### RNA sequencing and data analysis

3D4/21 cells were infected with ZJ01 PRV strain at a 0.5MOI; uninfected 3D4/21 cells were used as control. 22 h post-infection, cells were harvested by scraping and then put into Trizol (Invitrogen, USA). Three parallel replicates were performed for uninfected and infected cells. RNA isolation and sequencing were performed by Novogene Bioinformatics Technology Co., Ltd. (Beijing, China). Transcripts with a *P*-adjust < 0.05 were assigned as differentially expressed. GO and KEGG analyses were performed to understand the effect of PRV infection on cell biological processes, molecular function, and cellular components.

### RNA extraction and quantitative qRT-PCR

Total RNA was extracted from cells using a Total RNA Kit I (Omega Bioek). RNA purity was then detected by NanoDrop 2000 (Thermo Scientific, USA). The reverse transcription was performed using a HiScript II 1st Strand cDNA Synthesis Kit (Vazyme, China) following the manufacturer’s instructions. Quantitative RT-PCR was performed using AceQ ® qPCR SYBR ® Green Master Mix (Vazyme, China) according to the manufacturer’s instructions. The quantity of cDNA was 100 ng and the final concentration of primers was 0.2 μM. The qRT-PCR thermal conditions were 95 °C for 5 min, followed by 40 cycles of 95 °C for 10 s and 60 °C for 30 s, then 95 °C for 30 s, 60 °C for 60 s, 95 °C for 15 s. Data are presented as the fold change in gene expression normalized to β-actin and relative to the mock-infected control. Each reaction was performed in triplicate, and the data are calculated as the mean (M) ± SEM. The sequences of primer for genes are shown in Table 1.

**Table 1:** **Primer sequences used for qRT-PCR analysis**

Primer	Sequence (5′→3′)	Product
IFN-alpha-Fwd	TCCAGAAACCTGCAAGACAG	IFN-alpha
IFN-alpha-Rev	ATGGGCTTGTTAGTCTGTGAG	
IFN-beta-Fwd	ACCACAGCTCTTTCCATGAG	IFN-beta
IFN-beta-Rev	CAGGGACCTCAAAGTTCATCC	
IFN-gamma-Fwd	AATGGTAGCTCTGGGAAACTG	IFN-gamma
IFN-gamma-Rev	ACTTCTCTTCCGCTTTCTTAGG	
18s rRNA-Fwd	CGTTGATTAAGTCCCTGCCCTT	18s rRNA
18s rRNA-Rev	TCAAGTTCGACCGTCTTCTCAG	
U2snRNA-Fwd	CATCGCTTCTCGGCCTTTTG	U2snRNA
U2snRNA-Rev	TGGAGGTACTGCAATACCAGG	
gB-Fwd	GTCCGTGAAGCGGTTCGTGAT	PRV-gB
gB-Rev	ACAAGTTCAAGGCCCACATCTAC	
β-actin-Fwd	GTGATCTCCTTCTGCATCCTGTC	β-actin
β-actin-Rev	CTCCATCATGAAGTGCGACGT	
lnc641-Fwd	CAGGCATAGAGGGTTAAGGAC	lnc641
lnc641-Rev	ACGCTTTGCATGTGGAATTC	
ALD1114-Fwd	GGTGGGCAAAAGAACTTAGTG	ALD1114
ALD1114-Rev	GATAAGAACACGGCTCCCTG	
lnc1007-Fwd	CTCAGTGGGTTAATGATCCGG	lnc1007
lnc1007-Rev	CATATGGAGGTTCCCAGGTTAG	
ALD8954-Fwd	AAGTGGTACAAGACAGTGTGG	ALD8954
ALD8954-Rev	GGAGGTTGGAGGTAAAAGGAC	
lnc1059-Fwd	TCTTGGGCTCTGCAAATGAG	lnc1059
lnc1059-Rev	AAGGCTCCTTCTGTCTTGTTC	

### Western blot analysis

Cells were lysed on ice for 10 min in lysis buffer (Beyotime, China), then resolved by 10% SDS-PAGE and transferred onto nitrocellulose membrane. The membrane was blocked with 10% low-fat milk for 2 h at room temperature and then incubated with antibodies: anti-PRV gB-protein (1B1, 1:5000), anti-GAPDH (1:5000), anti-P-STAT1 (1:5000), anti-STAT1 (1:1000), anti-p-JAK1 (1:1000), anti-JAK1 (1:1000) for 2 h at room temperature. Membranes were incubated with HRP-conjugated goat anti-mouse and anti-rabbit IgG (H–L) secondary antibodies (1:1000; Beyotime, China). The proteins on the membranes were observed using the Chemistar High-sig ECL Western blotting substrate (Tanon, China) and developed on a Tanon 5200 system (Tanon, China).

### Indirect immunofluorescence assay (IFA)

PRV-infected cells were fixed with 4% paraformaldehyde for 15 min and then permeabilized with 0.1% Triton X-100. After three washes with PBS, cells were incubated with an anti-PRV gB-protein monoclonal antibody (1B1, 1:2500, made in our laboratory) for 1 h at 37 °C. Cells were washed three times with PBS and then incubated with Alexa Fluor 488-conjugated goat anti-mouse IgG (H–L) (1:200, Proteintech) for 1 h at 37 °C in the dark. Nuclei were stained with 4′,6-diamidino-2-phenylindole (DAPI; 100 ng/mL, Beyotime, Nanjing, China) for 5 min at room temperature. Immunofluorescence was observed using an inverted fluorescence microscope (Zeiss Axio Observer; Zeiss, Germany).

### Plasmid construction

Total RNA was extracted from 3D4/21 cells using a Total RNA Kit I (Omega Biotek, Norcross, GA, USA), and cDNA synthesis was performed with SuperScript III Reverse Transcriptase (Invitrogen). Lnc641 was generated by PCR amplification of cDNA from 3D4/21 cells with the oligonucleotide pair KpnI lnc641 5ʹ-CGC GGT ACC ATG CAA GGA CTG AGG GAG AGA GAG CGC CGA-3ʹ and XhoI lnc641 5ʹ-GCG CTC GAG CTA TGC ATG GCC ATG CAA GGA AAT CGG TGT T-3ʹ The sequence of the amplification product was compared to that in the transcriptome results for verification, restriction digested, and cloned into the pcDNA3.1(+) vector to produce pcDNA3.1-lnc641.

### Plasmid transfection and virus challenge

To determine the effects of lncRNA on PRV replication, 3D4/21 cells plated in 24-well plates were transfected with 0, 0.4, 0.8, 1 μg of pcDNA3.1-lnc641 using Lipofectamine 3000 (Invitrogen) according to the manufacturer’s recommendations. Twenty-four hours after transfection, the cells were infected with PRV (0.01 MOI) and then harvested for qRT-PCR, Western blotting and IFA at 24 hpi.

### Small interfering RNA assays

Three siRNAs targeting lnc641 were designed and synthesized by Invitrogen. The primer sequences used were as follows: siRNA1 (5′-GAC GAA CUU GAC AAG ACU AdTdT-3′, 5′-UAG UCU UGU CAA GUU CGU CdTdT-3′); siRNA2 (5′- GGA AGG CUA AGA AGG AGA AdTdT-3′, 5′-UUC UCC UUC UUA GCC UUC CdTdT-3′). 3D4/21 cells plated in 24-well plates were transfected with siRNAs or negative control (NC) using Lipofectamine 3000 Transfection Reagent (Invitrogen), following the manufacturer’s instructions. After 36 h, the cells were infected with PRV (0.01 MOI) for 24 h. Cells were harvested for qRT-PCR, TCID_50_, Western blot and IFA.

### Virus titration

3D4/21 cells grown in 96-well plates were infected with tenfold serial dilutions of PRV samples in four replicates. After 1 h at 37 °C, the culture medium was replaced with fresh DMEM. The plates were incubated for 72 h at 37 °C. The PRV titers were calculated using the Reed-Muench method.

### Isolation of cytoplasmic and nuclear RNAs

Cytoplasmic and nuclear fractions were prepared from 3D4/21 cells using PARIS™ Kit (Invitrogen, USA). cDNA was prepared using 1 μg RNA and qRT-PCR was performed to analyze both cellular fractions using primers of β-actin, lnc641, U2snRNA and 18sRNA. The expression of mRNA or lncRNA in nucleus and cytoplasm was calculated with the equation 2^−Δct^. The percentage of each RNA in the nucleus and cytoplasm was calculated.

### Statistical analyses

GraphPad Prism 7.0 software (GraphPad, La Jolla, CA, USA) was used to analyze all statistical data via one-way analysis of variance. Differences between two groups were considered statistically significant when the *P*-value was < 0.05, highly significant at *P* < 0.01, and extremely significant at *P* < 0.0001.

## Results

### LncRNAs are differentially expressed in PRV-infected 3D4/21 cells

**Figure 1:**
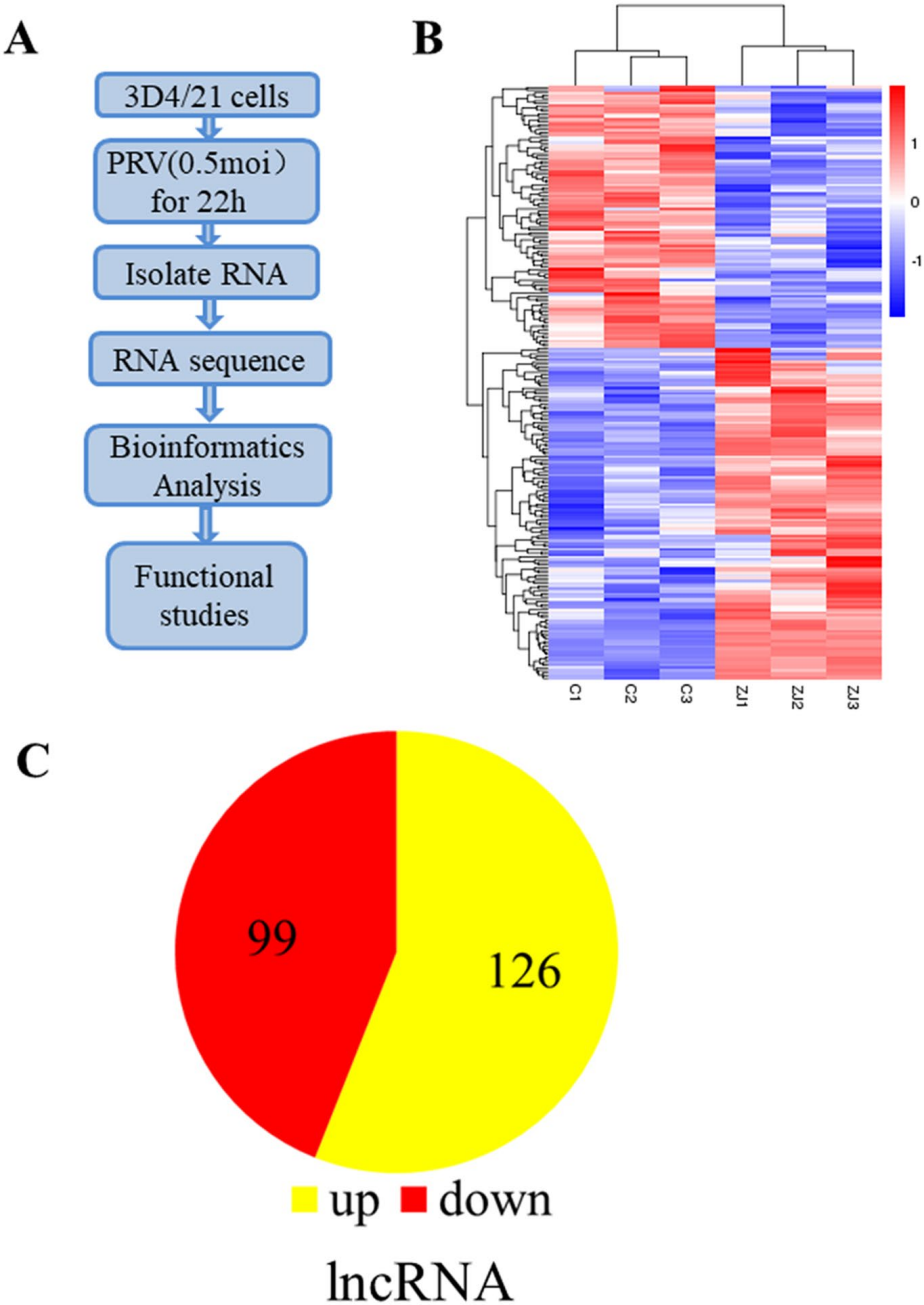
**LncRNAs are differentially regulated during PRV infection.** **A** Flowchart of RNA-seq experiment design. **B** Hierarchical clustering analysis of DEGs. DEG expression levels are represented as FPKM-normalized log_2_-transformed counts. Blue indicates low relative expression, and red indicates high relative expression. **C** Pie charts of significantly changed lncRNAs with a *P*-value of ≤ 0.05. Red and yellow colors denote downregulated and upregulated genes, respectively.

To identify lncRNAs that are dysregulated during Pseudorabies virus infection, 3D4/21 cells infected with ZJ01 PRV strain at a MOI of 0.5 for 22 h were submitted to RNA-seq analysis. Tophat was used for read mapping and Cufflinks/Cuffdiff was used for gene expression quantification. Figure 1A depicts the strategy of the experiment. Using a *P* value of < 0.05, 225 significantly differentially expressed lncRNAs were identified in PRV-infected 3D4/21 cells (Figure 1B). Of them, 126 1ncRNAs were upregulated and 99 1ncRNAs were downregulated (Figure 1C). There were 29 upregulated and 2 downregulated lncRNAs based on a fold change of 2 or more.

Five lncRNAs were chosen for further study as they were (a) a fold change of > 2 for upregulated lncRNAs, (b) significant changes in the expression of neighboring genes (up or down) within 10 000 kb of lncRNAs [22](Table 2).

**Table 2:** **Selected lncRNAs and their properties**

lncRNA_ID	lncRNA_Gene_ID	Gene_Type	Status	Fold change	Length (bp)
LNC_000641 (lnc641)	XLOC_037995	lincRNA	Novel_lncRNA	inf	385
LNC_001007 (lnc1007)	XLOC_063386	lincRNA	Novel_lncRNA	inf	685
LNC_001059 (lnc1059)	XLOC_067112	antisense_lncRNA	Novel_lncRNA	inf	1559
ALDBSSCT0000011114 (ALD11114)	ALDBSSCG0000006748	lincRNA	Annotated_lncRNA	inf	316
ALDBSSCT0000008954 (ALD8954)	ALDBSSCG0000005482	lincRNA	Annotated_lncRNA	3.8547	4599

**Figure 2:**
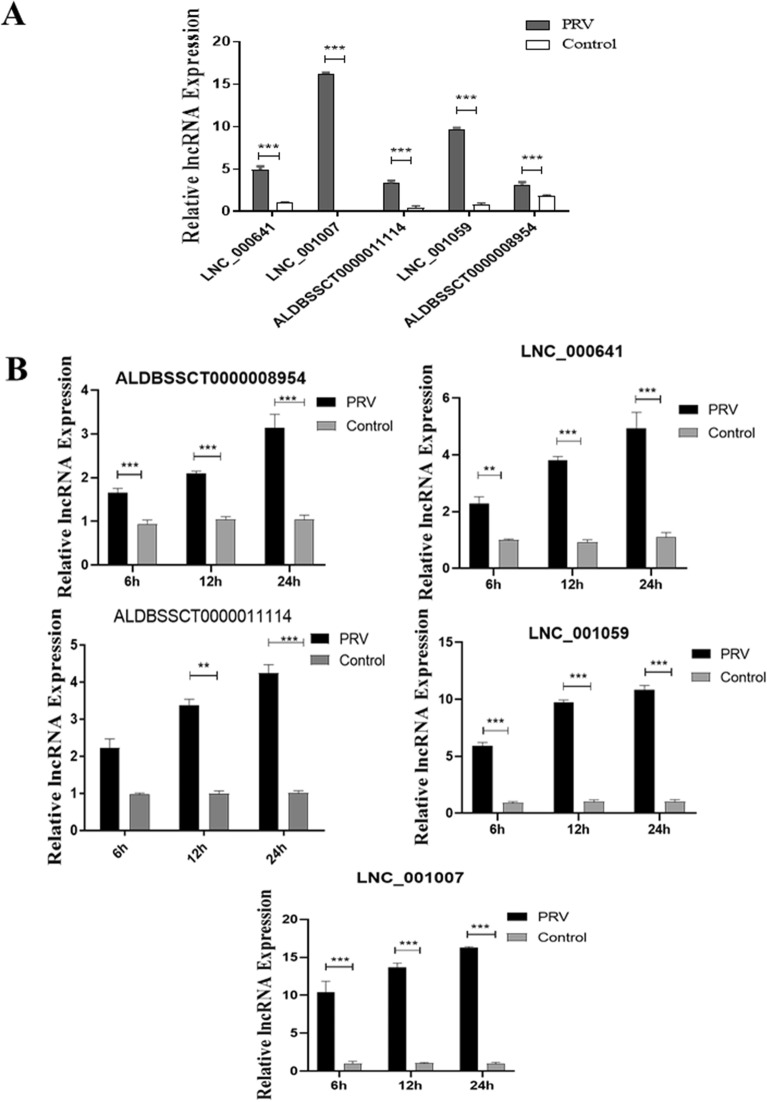
**Validation of RNA-seq results with qRT-PCR.** **A** 3D4/21 cells were infected with ZJ01 PRV strain at a MOI of 0.5 for 22 h. Relative expression levels of selected lncRNAs were determined by qRT-PCR and normalized to β-actin. **B** qRT-PCR quantification of the expression levels of lncRNA at three time points (6 h, 12 h, 24 h) after PRV infection. The qRT-PCR was repeated at least three times, with each experiment performed in triplicate. ***P < 0.001; **P < 0.01; *P < 0.05 vs mock control cells for each time point.

The qRT-PCR was then utilized to validate the results from RNA-seq analysis. The qRT-PCR confirmed that the production of all these 5 lncRNAs were promoted by ZJ01 (Figure 2A). To further confirm the results of the RNA-seq analysis, a qRT-PCR assay was conducted to measure the expression of lncRNA in PRV-treated cells at 6 h, 12 h, 24 h. The results showed that the expression levels of 5 groups of lncRNA increased along with PRV infection (Figure 2B). Taking together, these data indicated that PRV infection of 3D4/21 cells promoted the above 5 lncRNA production, consistent with the RNA sequencing results.

### Effects of different PRV strains and cell lines on lncRNA expression

**Figure 3:**
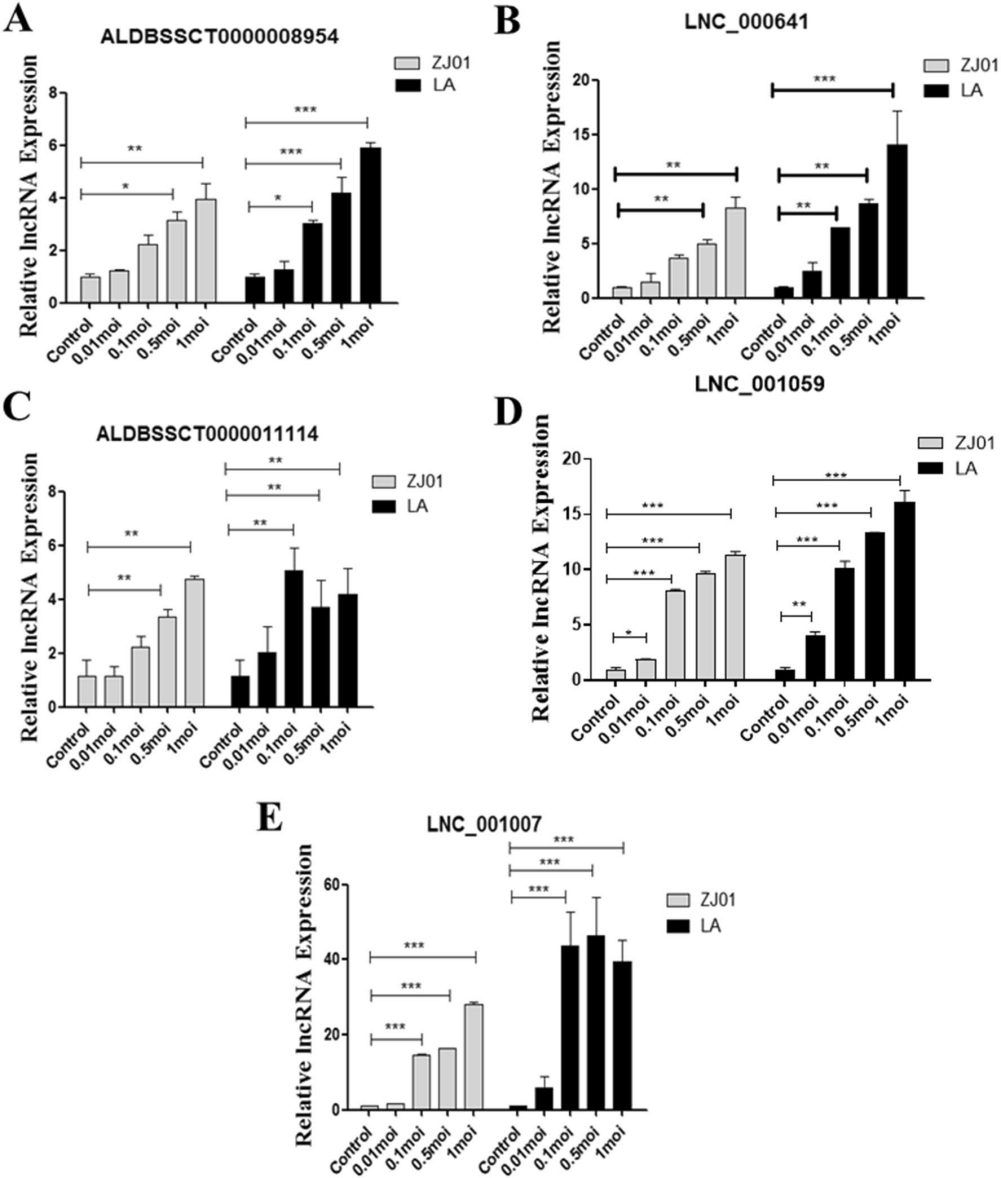
**LncRNA induction by different PRV strains.** 3D4/21 cells were infected with two PRV strains, ZJ01 (MOI: 0.01, 0.1,0.5 and 1), LA (MOI: 0.01, 0.1,0.5 and 1) for 24 h. LncRNA expression levels were determined by qRT-PCR and normalized to β-actin. Results are represented as means ± SEM from three independent experiments. *p < 0.05, **p < 0.01 and ***p < 0.001 vs. mock control.

In RNA sequencing analysis, a single MOI of ZJ01 PRV strain was used to infect 3D4/21 cells. To study the effect of doses and strains of PRV on the upregulated IncRNAs, 3D4/21 cells infected with different doses of PRV ZJ01 and LA strains were used to detect the lncRNAs productions. The result showed that the lncRNAs could be produced by both ZJ01 and LA strains in a dose-dependent manner (Figure 3). However, the magnitude of induction varied among strains and lncRNAs. ALD11114 expression reached maximum at a MOI of 0.1 and lnc1007 expression reached maximum at a MOI of 0.5 while ALD8954, lnc641, and lnc1059 had a highest expression at a MOI of 1. LA strain induced higher levels of 5 lncRNAs than ZJ01 strain.

**Figure 4:**
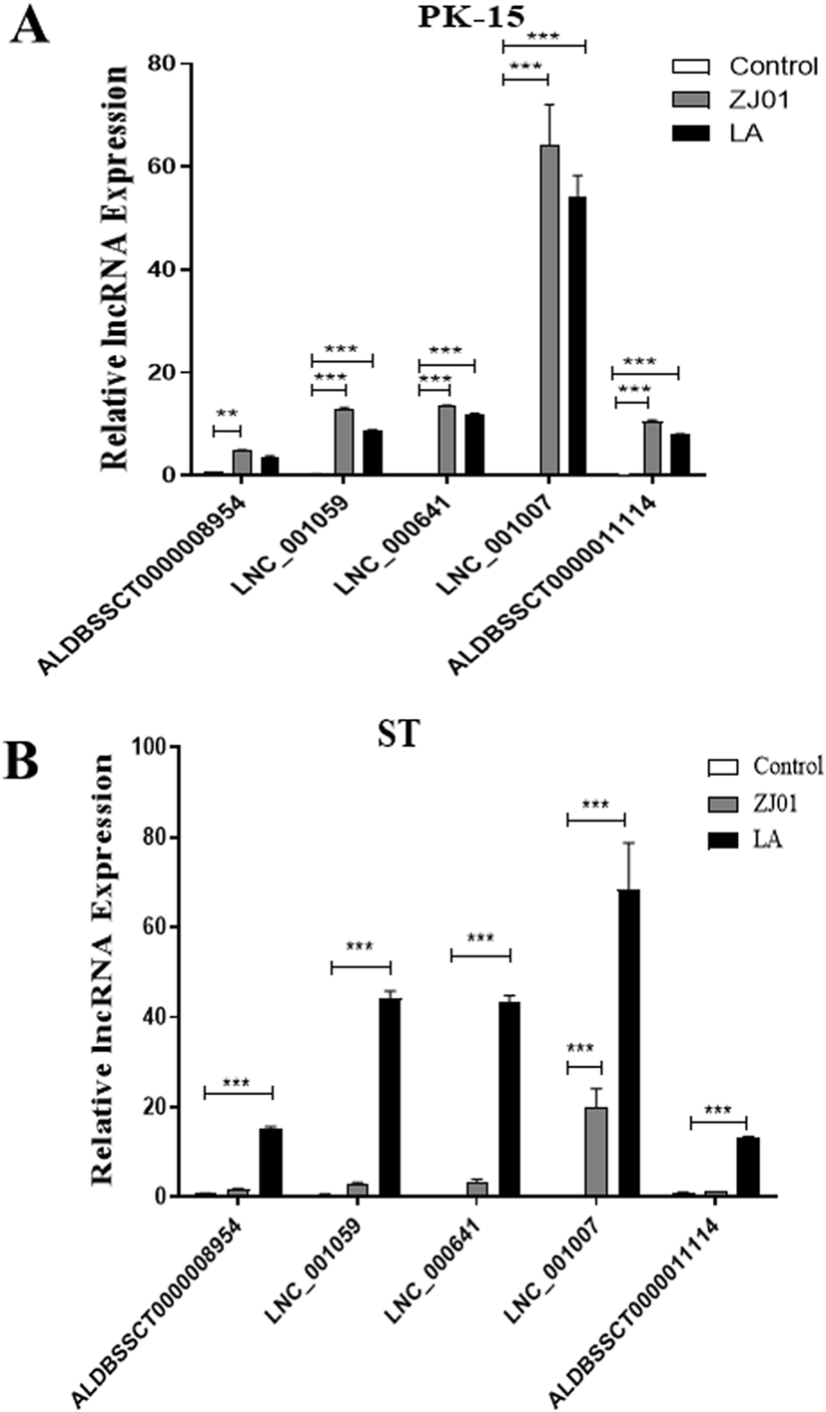
**Lnc641 induction in PK-15 and ST cells by PRV.** PK-15 and ST cells were infected with ZJ01 and LA PRV strains at a MOI of 0.5 for 22 h. LncRNA expression levels were determined by qRT-PCR and normalized to β-actin. The results are represented as means ± SEM from three independent experiments. *p < 0.05, **p < 0.01 and ***p < 0.001 vs. mock control.

To determine whether lncRNA was also produced in other cells, Porcine kidney (PK-15) cells, Porcine testis (ST) cells infected with 0.5 MOI of ZJ01 or LA for 24 h. The results showed that the induction was more potent in ST cells than in PK-15 cells (Figure 4).

### Knockdown of lnc641 by siRNAs inhibits PRV replication

**Figure 5:**
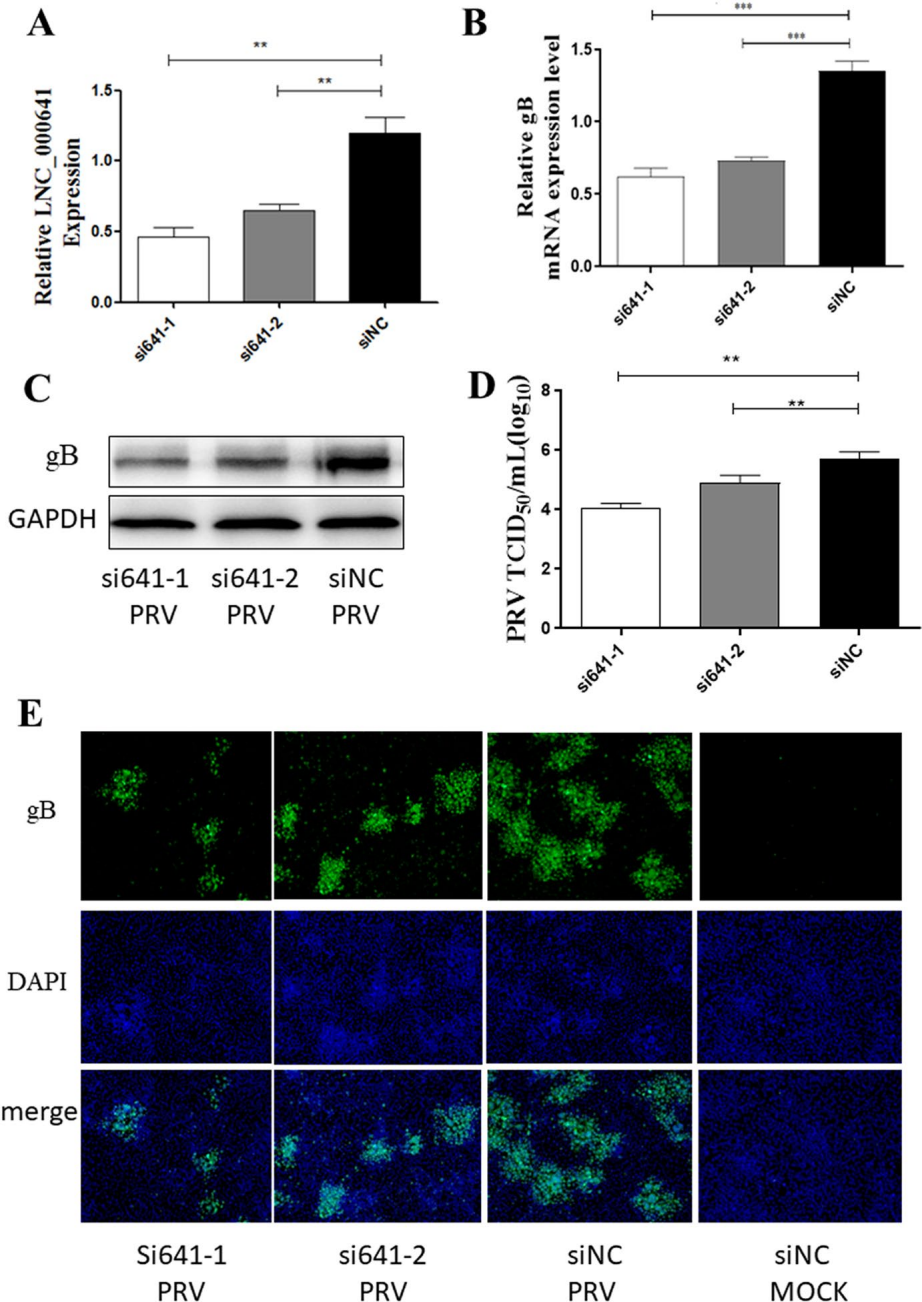
**Knockdown of lnc641 by siRNAs inhibits PRV replication.** **A** 3D4/21 cells were transfected with two siRNAs (si641-1, si641-2) or negative control (siNC). At 36 h post-transfection, the knockdown efficiency of lnc641 was determined by qRT-PCR. **B**–**E** 3D/21 cells placed in 24-well plates were transfected with the two siRNAs for 36 h and then infected with ZJ01 (0.01 MOI). After 24 h, the cell samples were collected to measure the replication of PRV by qRT-PCR (**B**) and Western blot assays (**C**). The supernatant was used to measure the viral titers by TCID_50_ analysis (**D**). The cells were treated as described previously, and IFA was performed with a primary anti-gB protein monoclonal antibody to analyze the antiviral effect of PRV. Viral gB-protein is green, and nuclei are blue (**E**). Results are presented as means ± SEM of data from three independent experiments. * *P* value < 0.05, ** *P* < 0.01, *** *P* < 0.0001.

The lnc641 with the highest fold changes were selected for further characterization and functional studies. To determine the functional role of lnc641 in PRV replication, 3D4/21 cells were transfected with two siRNAs designed to be targeting lnc641 gene. After incubation for 36 h, lnc641 RNA levels were detected by qRT-PCR (Figure 5A). The results showed that siRNA1 and siRNA2 significantly downregulated lnc641 RNA levels in 3D4/21 cells. Then 3D4/21 cells were transfected with the siRNA, and then infected with ZJ01 strain at 0.01 MOI at 24 h post-transfection. At 24 hpi, cells were harvested for TCID_50_, qRT-PCR, Western blot assay and IFA. As shown in Figures 5B–E, the replication of PRV and the expression of gB were reduced to a certain extent when siRNA knocked down lnc641, compared with the cells transfected with negative control siRNA (siNC). TCID_50_ assays also showed that the knockdown of lnc641 significantly decreased viral titers.

### Overexpression of lnc641 enhances PRV replication

**Figure 6:**
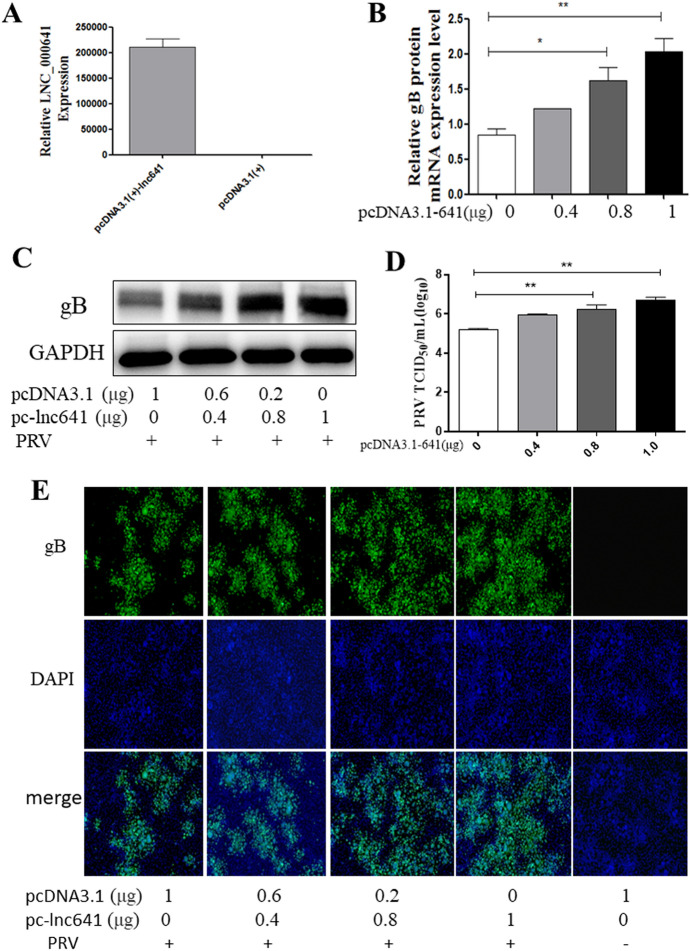
**Lnc641 overexpression enhances PRV replication.** **A** 3D4/21 cells were transfected with 1 μg pcDNA3.1(+)-641 or pcDNA3.1(+). At 24 h post-transfection, the overexpression efficiency of lnc641 was determined by qRT-PCR. **B**–**E** 3D4/21 cells were transfected with the indicated doses of pcDNA3.1(+)-641 or pcDNA3.1(+) for 24 h, followed by infection with ZJ01 (0.01 MOI) for 24 h. The viral gB protein and mRNA levels were evaluated by qRT-PCR (**B**) and Western blot (**C**). The supernatant was used to measure viral titers by TCID_50_ analysis (**D**). The cells were treated as described previously, and IFA was performed with a primary anti-gB protein monoclonal antibody to analyze the antiviral effect of PRV. Viral gB-protein is green, and nuclei are blue (**E**). Results are presented as means ± SEM of from three independent experiments. *P value < 0.05, **P < 0.01, ***P < 0.0001.

To confirm the effect of lnc641 on PRV replication, 3D4/21 cells were transfected with different doses of pcDNA3.1-lnc641 or pcDNA3.1(+) and then infected with ZJ01 (0.01 MOI). At 24 hpi, cells were harvested for PRV detection by TCID_50_, Western blot, qRT-PCR and IFA. The results showed that overexpression of lnc641 significantly enhanced PRV replication (Figures 6A–E).

### Lnc641 regulates PRV replication by inhibiting type I interferon

**Figure 7:**
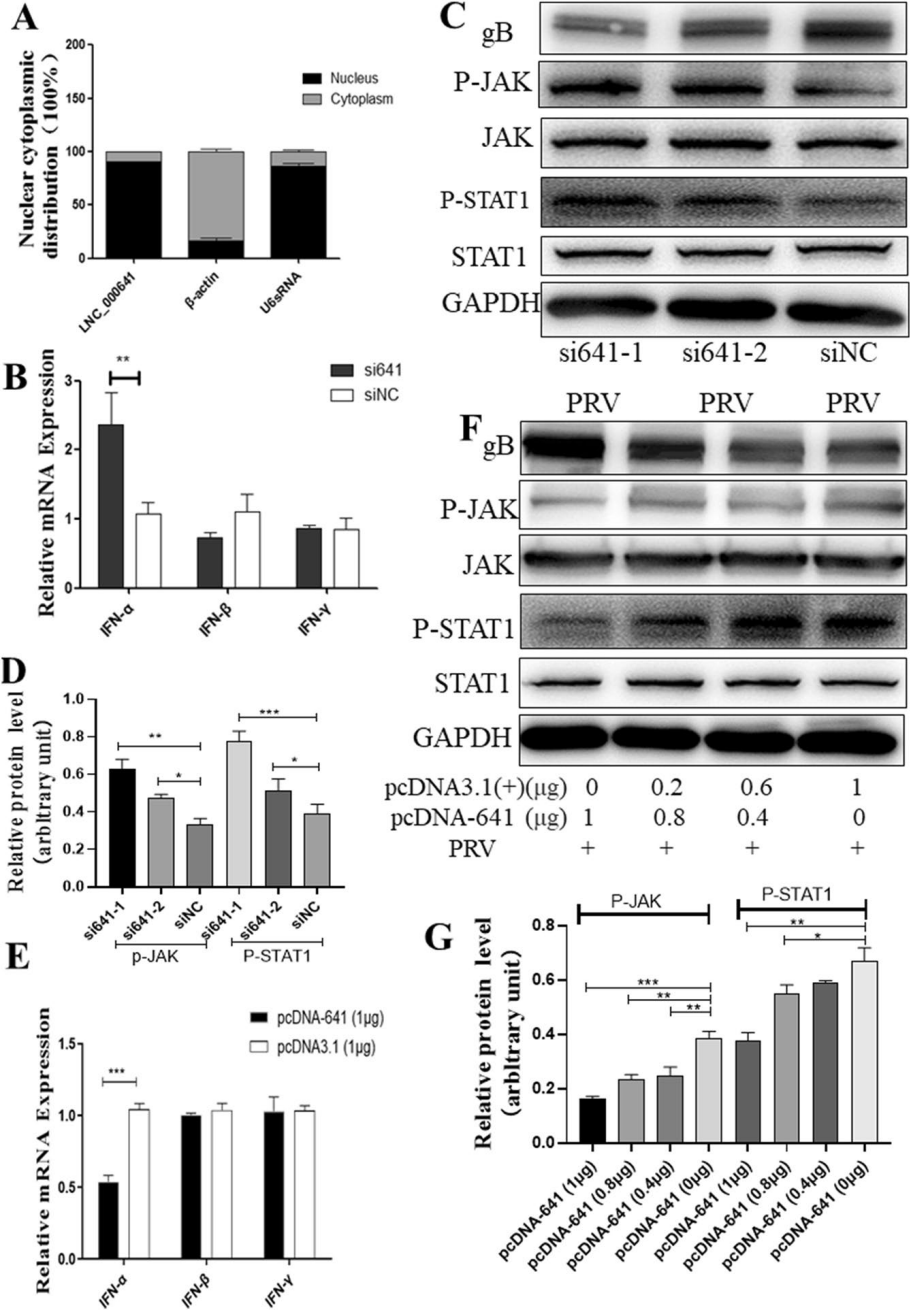
**Lnc641 regulates PRV replication by inhibiting type I interferon.** **A** The levels of lnc641, β-actin mRNAs (cytoplasmic RNA positive controls), and U2snRNA (nuclear RNA positive control) in cytoplasmic and nuclear fractions of 3D4/21 cells were determined by qRT-PCR. Results are represented for each gene as means ± SEM from three independent experiments. **B**, **C** 3D4/21 cells transfected with si641 and siNC for 36 h, then cells were infected with ZJ01 (0.01 MOI). After 24 h, IFN mRNA levels were detected by qRT-PCR. Meanwhile, the levels of JAK and STAT1 protein were detected by Western blot. **D** Use of Image Quant software to quantify the protein bands in Fig. 7C Western blot, as normalized to GAPDH. **E** 3D4/21 cells were transfected with 1 μg pcDNA3.1(+)-641 or pcDNA3.1(+) for 24 h, followed by infection with ZJ01 (0.01 MOI) for 24 h. Then IFN mRNA levels were detected by qRT-PCR. **F** 3D4/21 cells were transfected with the indicated doses of pcDNA3.1–641 or pcDNA3.1(+) for 24 h, followed by infection with ZJ01 (0.01 MOI) for 24 h, the levels of JAK and STAT1 protein were detected by Western blot. **G** Use of Image Quant software to quantify the protein bands in **F** Western blot, as normalized to GAPDH.

To elucidate the mechanisms of lnc641 mediated PRV replication, the location of lnc641 in cells was determined. The cytoplasmic and nuclear fractions from 3D4/21 cells were isolated and lnc641 levels in both fractions were determined by qRT-PCR. As shown in Figure 7A, lnc641 was enriched in the nucleus as nuclear U2snRNA, a positive control gene. Cytoplasmic β-actin mRNAs were primarily located in the cytoplasm.

To examine the role of lnc641-induced IFN expression in PRV infection, 3D4/21 cells were respectively transfected with siRNA1, siRNA2 and negative control siRNA (siNC) for 36 h and then infected with ZJ01 (0.01 MOI) for 24 h, followed by qRT-PCR for IFN mRNA (Figure 7B) and Western blot for JAK/STAT protein (Figures 7C, D). The results showed that after lnc641 silencing, only IFN-alpha mRNA expression increased significantly, but not IFN-beta and IFN-gamma. At the same time, knocking down of lnc641 increased the phosphorylation of JAK and STAT1 proteins.

PRV infection suppresses IFN-induced upregulation of a subset of ISGs and STAT1 phosphorylation, indicating an impairment of IFN signaling in PRV-infected cells [23]. To confirm the effect of lnc641-induced IFN expression on PRV infection, 3D4/21 cells were transfected with pcDNA3.1–641 or pcDNA3.1(+) for 24 h and then infected with ZJ01 (0.01 MOI) for 24 h, followed by qRT-PCR for IFN mRNA (Figure 7E) and Western blot for JAK/STAT1 protein (Figures 7F, G). The results showed that lnc641 overexpression inhibited the IFN-alpha mRNA and reduced the phosphorylated expression of JAK and STAT1.

## Discussion

Pseudorabies virus is an important pathogen in the swine industry. In addition to proteins encoded by viruses, non-coding RNAs including microRNAs and lncRNAs in host cells may play an important role in viral infections [24–26]. LncRNAs represented a potential class of host factors and will be new alternatives for development of host-centered antiviral strategies. However, there were few reports on the interaction mechanism between PRV and lncRNAs [27, 28]. It was well known that lncRNAs play an important role in virus invasion and the corresponding antiviral immune response. Macrophages also played an important role in the first line of defense against pathogens invading the body. In this study, the porcine alveolar macrophage cells (3D4/21) infected with ZJ01PRV strain was used in RNA-Sequencing to identify differential lncRNAs. A total of 2424 lncRNAs were screened, of which 1320 were unannotated lncRNAs. 225 were significantly changed. Among the 126 significantly upregulated lncRNAs in ZJ01-infected cells, 5 lncRNAs were further studied. The results showed that they also induced by other another PRV strain, LA. However, the magnitude of induction of the same lncRNAs varied between ZJ01 and LA, indicating that there were differences between virus strains in induction of lncRNAs expression. Compared with the traditional strain LA, some genes of the variant strain ZJ01 were mutated. This may account for the different inducibility of lncRNA between ZJ01 and LA. But the specific genes that affect the inducibility need to be further studied. In this study, much lower MOI (0.01 and 0.5) were applied for virus infection which were entirely different from the usual doses used in different experiments. This was related to the virulence of the virus and the effect of transfection agents on cells.

LncRNAs play an important regulatory role in the battle between virus and host, involving the transcription of viral and host genes, stability and translation of mRNAs, and host antiviral response [29, 30]. Host cells can initiate an antiviral response after viral infection by altering their own lncRNA expression. In response to virus infections, lncRNAs have been shown to modulate virus infections by diverse mechanisms [31, 32]. In this study, a lncRNA named lnc641 has been shown to promote PRV replication.

The location of lncRNA in the cell may provide important information on how to achieve its function. The lncRNA in the cytoplasm such as DANCR can compete for microRNA binding sites [33]. Another nuclear lncRNA, NRAV functions as a histone modification factor of anti-viral genes, MxA and IFITM3 [15]. The study proved that lnc641 was mainly distributed in the nucleus of 3D4/21 cells. Recently, an increasing number of lncRNAs has been reported to play roles in the innate immune response to virus infections [34, 35]. Previous studies have implicated miR‐155 in host immunity against viral infections and regulation of type I IFN signaling [36, 37]. It is known that lncRNA Malat1 can inhibit the production of type I IFN in macrophages after virus infection [38], and lncRNA Sros1 can promote IFN-γ–STAT1 mediated innate immunity [39]. Knockdown of lncRNA NONMMUT057981 can promote VSV-induced IFN production in mouse peritoneal macrophages [13]. In this study, the results demonstrated that the production of IFN-alpha and the phosphorylation of JAK and STAT1 could be regulated by knockdown and overexpression of lnc641, which indicated that lnc641 promoted the replication of PRV by regulation of IFN-alpha though JAK/STAT1 pathway. However, how lnc641 regulates the changes of JAK/STAT1 and IFN needs further research in the future.

In summary, this study determined that the lnc641 was significantly induced by PRV infection and has a profound effect on PRV replication in vitro. In addition, the results indicated that the lnc641 inhibits the innate immune response to PRV infection by down-regulating the production of IFN-α by inhibiting the JAK/STAT1 pathway, thereby increasing the replication of PRV. Integrated analysis showed that differentially expressed lncRNA may play a critical role in regulating PRV replication, and may provide new insights for PRV prevention and treatment strategies in the future. To illustrate the effect of lncRNAs on PRV infection, other lncRNAs will be analyzed in the following studies.

## Data Availability

All data generated or analyzed during this study are included in this published article.
